# Influence of mandatory generic substitution on pharmaceutical sales patterns: a national study over five years

**DOI:** 10.1186/1472-6963-8-50

**Published:** 2008-02-29

**Authors:** Karolina A Andersson, Max G Petzold, Peter Allebeck, Anders Carlsten

**Affiliations:** 1 Social Medicine, Department of Public Health and Community Medicine, the Sahlgrenska Academy at Göteborg University, Göteborg, Sweden; 2 Nordic School of Public Health, Göteborg, Sweden; 3 Statistical research unit, Göteborg University, Göteborg, Sweden; 4 Division of Social Medicine, Department of Public Health Sciences, Karolinska Institute, Stockholm, Sweden; 5 Department of Research and Development, The National Corporation of Swedish Pharmacies (Apoteket AB), Göteborg, Sweden

## Abstract

**Background:**

Mandatory generic substitution was introduced in Sweden in October 2002 in order to try to curb escalating pharmaceutical expenditure. The aim of this study was to investigate how sales patterns for substitutable and non-substitutable pharmaceuticals have developed since the introduction of mandatory generic substitution; furthermore, to compare sales patterns in different groups of the population, based on patients' age and gender.

**Methods:**

Five therapeutic groups comprising both substitutable and non-substitutable pharmaceuticals were included. The study period was from January 2000 to June 2005. National sales data were used, covering volumes of dispensed prescription medicines (expressed in defined daily doses per 1000 inhabitants and day) of each pharmacological substance in the therapeutic groups for each age and gender group. Sales patterns for substitutable and non-substitutable pharmaceuticals were compared using a descriptive approach.

**Results:**

In most therapeutic groups there has been an increase in the volumes of substitutable pharmaceuticals sold since the introduction of the reform, ranging from one third to three times the initial volume; whereas the volumes of non-substitutable pharmaceuticals have levelled out or declined. There were few gender differences in sales patterns of substitutable and non-substitutable drugs. In three therapeutic groups, sales patterns differed across different age groups, and there was a tendency for volumes of recently introduced non-substitutable pharmaceuticals to be proportionally higher in the youngest age groups.

**Conclusion:**

Since the introduction of the reform, there has been a proportionally larger increase in sales of substitutable pharmaceuticals compared with sales of non-substitutable pharmaceuticals. This indicates that the reform might have contributed to larger sales of less expensive pharmaceuticals.

## Background

Most countries promote the use of generic drugs, and several countries have introduced generic substitution in order to increase the use of generics [1-6]. Generic substitution has contributed to increased use of generic brands and to cutting the escalating costs in several countries [2,3,6]. Some studies have shown that physicians were reluctant to generic substitution policies [7-9]. There have been concerns that physicians might perceive it as a threat to their autonomy, and would thus oppose such policies. An Australian study reported that physicians more frequently switched therapy for patients using non-substitutable drugs than for patients using substitutable drugs, i.e. pharmaceutical products containing the same active ingredient with the same strength [5]. The study showed that therapy was changed in a small proportion of the patients. The authors of the study noted that possible explanations for the low frequency of switched therapy include a clear price signal for the investigated products contributed to cost-consciousness among prescribers and patients; and that prescribers were aware of the fact that problems following changed therapy [5].

In order to increase price competition for medically equivalent products where the patent had expired, Sweden introduced mandatory generic substitution on 1 October 2002 [10,11]. Prescriptions issued on or after 1 October 2002 were covered by the reform. Prescriptions are valid for one year in Sweden and thus the reform was fully implemented a year after the introduction. According to the Swedish Pharmaceutical Benefits Scheme, PBS, patients' expenses/co-payments for reimbursed medicines are accumulated during twelve-month periods, starting on the day of the first purchase. The patient co-payment corresponds to the price of the drugs within the scheme until the accumulated costs have reached a certain level and thereafter a decreasing proportion of the price until the high-cost threshold has been reached. After the high-cost threshold there is no co-payment for reimbursed pharmaceuticals during the rest of the period. The Swedish Medical Products Agency produces a list of products that are substitutable, based on the judgement that they are medically equivalent. To be considered as medically equivalent the products must contain the same active pharmacological substance, of the same strength and in a comparable package size. According to the law on generic substitution, pharmacy personnel are obliged to offer the patient the cheapest available substitutable drug according to the MPA's list, unless substitution is restricted [10]. Prescribers can restrict substitution for medical reasons by marking 'substitution not allowed' on the prescription. Substitution can also be restricted for other reasons: e.g. divided doses, such as divided tablets, potential reactions to non-active ingredients or differences in taste. The pharmacist makes this decision. If substitution is restricted by the physician or the pharmacist, the total cost of the prescribed drug is added to the patient's accumulated cost of drugs purchased within the PBS. The patient can oppose substitution and retain a more expensive product, given that he or she pays the price difference between the prescribed and cheapest (reimbursed) product out of pocket. In this case, the cost of the cheapest product is added to the patient's accumulated cost of reimbursed drugs if the patient has not yet reached the high-cost threshold. The price difference is paid out of pocket, irrespective of the patient's co-payment status.

The aim of the reform was to reduce costs for off-patent pharmaceuticals, through increased price competition, as well as substituting with cheaper equivalents. The introduction of generic substitution was followed by notable reductions in price for several top-selling products, which were highlighted in the media and elsewhere. These price cuts helped to curb the increase of overall pharmaceutical expenditure in Sweden after the introduction of generic substitution [12]. Parallel with the introduction of generic substitution, decentralisation of the responsibility for costs of pharmaceuticals within the PBS, from the government to the county councils, was in progress [13,14]. The decentralisation was initiated in 1998, continued in 2002 and fully implemented in January 2005. The responsibility of providing care to citizens lies with the county councils. County councils have had budget responsibility for inpatient drugs for many years. The aim of the shift of the budget responsibility was to integrate outpatient drug costs in the healthcare budget and make prescribers more cost-conscious. Today, this budget responsibility is handled at a central level in some county councils, whereas in some it has been decentralised to the care-unit level. The process has started a debate regarding cost issues of pharmaceuticals among physicians.

The introduction of generic substitution implied considerable changes in the prescribing process for prescribers and patients. The prescribers had to inform the patient that the pharmacy might substitute the prescribed drug with a cheaper equivalent and ensure that reports on performed substitutions sent by the pharmacies were documented in the patient records. The patients faced a new situation when asked to make decisions about substitution themselves; previously the prescribers had dealt with this. Investigating how the sales patterns of prescribed drugs have developed following the reform indicates whether the pattern of prescribing has changed since the reform was introduced. Earlier studies on effects of pharmaceutical reimbursement policies have shown differences between overall effects and effects in subgroups of the population. A previous study reported that although increased cost-sharing was not associated with negative effects on health status, decreased use of essential pharmaceuticals or hospital admissions on an overall level a negative impact on these outcomes was observed for particularly vulnerable groups such as the elderly and the poor [15-17]. Investigating how the sales patterns have developed in different age and gender groups could indicate whether the reform affected these groups differently.

The present study aims to investigate how the sales patterns for substitutable and non-substitutable pharmaceuticals have developed since the introduction of mandatory generic substitution in Sweden.

More specifically the objectives were:

1) to assess how volumes sold of substitutable pharmaceuticals, compared with volumes sold of non-substitutable pharmaceuticals, have changed since the reform was introduced

2) to investigate whether sales patterns vary between different groups of the population based on patients' age and gender

## Methods

Five therapeutic groups were selected for case studies. Selection criteria were that the therapeutic groups should encompass both substitutable and non-substitutable pharmaceuticals, be widely used in outpatient care and generate a substantial cost to society. Therapeutic groups used both for short- and long-term treatments were included (long-term treatment > 6 months) with an emphasis on long-term treatment. Pharmaceuticals were classified as substitutable if the patent had expired and one or more equivalent products were available. Table 1 shows the five therapeutic groups as well as the pharmacological substances of each therapeutic group, their status regarding substitutability and when substances became substitutable. In general, a pharmaceutical becomes substitutable when generic products have been introduced after patent expiry. Three therapeutic groups mainly used for long-term treatment were included. These were: 1) ACE-inhibitors (antihypertensives), 2) selective serotonin reuptake inhibitors, SSRIs (antidepressants) and 3) HMG CoA-reductase inhibitors, referred to as statins (cholesterol and triglyceride reducers). One group mainly used for short-term treatment, 4) nucleosides and nucleotides (antiviral), and one group used both for short- and long-term treatment, 5) proton pump inhibitors, PPIs (antiulcer agents), were also included. In general, the groups comprised top-selling substances covering both statuses of substitutability. For the two top-selling ACE-inhibitors, enalapril and ramipril, the situation was somewhat different. Generic versions of enalapril became available in the beginning of 2000. However, for ramipril there were only two alternatives, both were branded products produced by different premium brand companies, available until 2004 when generics became available. These were considered substitutable from October 2002 although there was no price competition.

**Table 1:** Pharmacological substances included in the selected therapeutic groups and their substitution status.

Therapeutic group	ATC-code	Substance	Substitutable	Substitutable since*
**Long-term treatments**				
ACE-inhibitors	C09AA	Captopril	Yes	1 October 2002
		Enalapril	Yes	1 October 2002
		Lisinopril	Yes	1 October 2002
		Ramipril	Yes	1 October 2002
		Quinapril	No	
		Cilazapril	No	
		Fosinopril	No	
		Trandolapril	No	
Selective Serotonin Reuptake Inhibitors, SSRI	N06AB	Fluoxetine	Yes	1 October 2002
		Paroxetine	Yes	1 October 2002
		Citalopram	Yes	1 October 2002
		Sertraline	No	
		Fluvoxamine	No	
		Escitalopram	No	
HMG CoA-reductase inhibitors, statins	C10AA	Fluvastatin	Yes	5 February 2003
		Simvastatin	Yes	28 March 2003
		Pravastatin	No	
		Atorvastatin	No	
		Rosuvastatin	No	
**Short-term treatments**				
Nucleosides and nucleotides	J05AB	Aciclovir	Yes	1 October 2002
		Ganciclovir	No	
		Famciclovir	No	
		Valaciclovir	No	
		Valganciclovir	No	
**Both short- and long-term treatments**				
Proton Pump Inhibitors, PPI	A10BC	Omeprazole	Yes	28 March 2003
		Lansoprazole	No	
		Pantoprazole	No	
		Esomeprazole	No	
		Rabeprazole	No	

* The date when the substance became substitutable according to the Swedish Medical Products Agency given for substitutable substances.

Sales data for the selected therapeutic groups comprising dispensed prescription pharmaceuticals encompassed by the PBS were included in the study. The study period covered pharmaceuticals dispensed between 1 January 2000 and 30 June 2005. Sales data of dispensed prescribed medicines (i.e. regular prescriptions and multidose-dispensed drugs) in outpatient care for the substances in the selected therapeutic groups were collected from the National Corporation of Swedish Pharmacies (Apoteket AB). Monthly sales data were collected that encompassed Sweden in total. Data comprised volumes sold, expressed as defined daily doses (DDD) with information on both substance and product as well as information on age, gender and place of residency of the patients collecting the dispensed drugs. Defined daily doses were constant over the study period. Age was divided into six categories: 0–4, 5–14, 15–44, 45–64, 65–74 and 75-years old. Due to few observations, no separate examinations were undertaken of the two youngest age categories.

The volumes of substitutable and non-substitutable substances were combined into two groups (denoted substitutable pharmaceuticals and non-substitutable pharmaceuticals) for comparison. Sales of non-substitutable packages, for example when only one company produces a certain package-size or strength, of substitutable substances were removed from the sales of that substance. Defined daily doses per 1000 inhabitants and day (DDD/tid) were calculated both for total volume of the substances, each substance and specifically for each age and gender group. Data on both total volumes of substitutable and non-substitutable pharmaceuticals as well as each substance were presented graphically. The sales patterns of dispensed drugs in the different subsets were compared with a descriptive approach, in order to investigate whether the sales pattern changed when the reform was implemented. The study was approved by the Ethics Committee at Göteborg University.

## Results

### Sales patterns of substitutable and non-substitutable drugs

**Figure 1:**
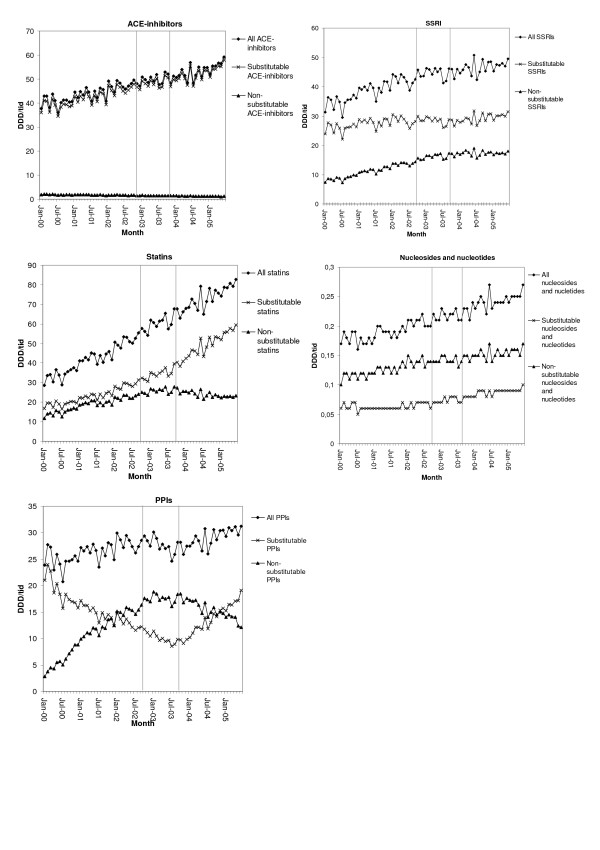
**Total sales volumes of non-substitutable and substitutable drugs in the included therapeutic groups**. Volumes expressed as defined daily doses per 1000 inhabitants and day (DDD/1000 inhabitants and day). The two vertical reference lines indicate the implementation period for generic substitution (1 October 2002–30 September 2003).

A comparison of the total volumes of substitutable and non-substitutable pharmaceuticals in the three therapeutic groups mainly used for long-term treatment (ACE-inhibitors, SSRIs and statins) showed that the volumes of substitutable pharmaceuticals were proportionally higher throughout the period (Figure 1). As shown in Figure 1, the volumes of substitutable statins tripled over the study period, whereas the volumes of non-substitutable statins doubled before the introduction of the reform and decreased somewhat thereafter. For the group mainly used for short-term treatment, nucleosides and nucleotides, the overall volume increased over the period. The sales of non-substitutable pharmaceuticals were proportionally higher than the sales of substitutable pharmaceuticals in this group. Until July 2003 there was a decrease in the volumes of substitutable PPIs, used both for short- and long-term treatment, to half of the initial volume when omeprazole became substitutable. Thereafter the volume increased and at the end of the study period it had almost reached its initial level. For non-substitutable PPIs, the pattern was the opposite.

There was an apparent change in the sales pattern for statins and PPIs during the year of implementation of generic substitution that was not seen in the other groups. The sales patterns for two of the selected groups, ACE-inhibitors and nucleosides and nucleotides, were unaffected by the reform.

**Figure 2:**
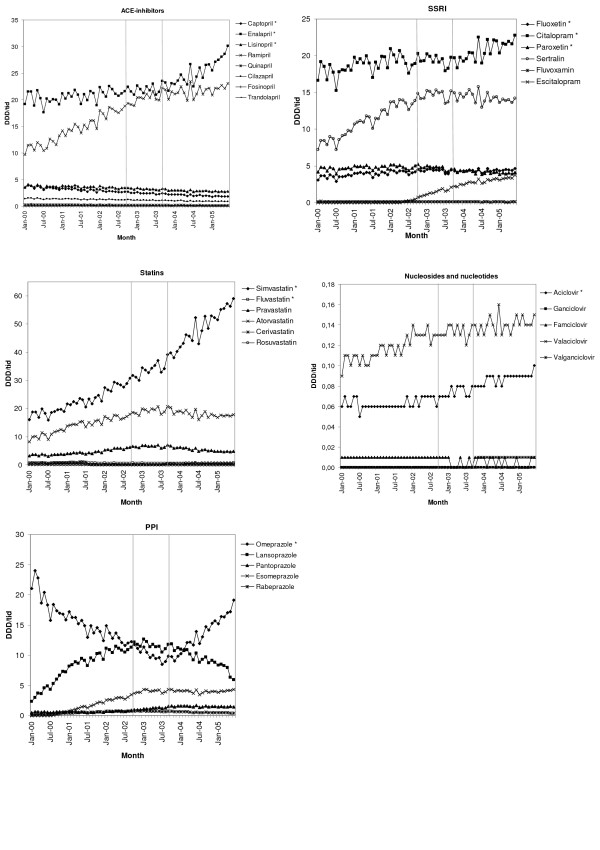
**Volumes sold of each substance in the included therapeutic groups**. Volumes sold for each substance included in the selected therapeutic groups, expressed as defined daily doses per 1000 inhabitants and day (DDD/1000 inhabitants and day). The two vertical reference lines indicate the implementation period for generic substitution (1 October 2002–30 September 2003). An * after the substance name indicates a substitutable substance.

As shown in Figure 2, sales of substances that became substitutable after the reform was introduced, constitute the largest volumes in all groups with the exception of the nucleosides and nucleotides. Top-selling non-substitutable substances have levelled out or decreased in four out of five groups since the reform was introduced. The sales pattern of the ACE-inhibitors has been somewhat different, as the two largest products are both substitutable. There has been a proportionally larger increase in the volumes of enalapril compared with ramipril since the introduction of the reform.

### Sales patterns of substitutable and non-substitutable drugs by age and gender

The volumes of dispensed prescription pharmaceuticals varied across different age groups in the selected therapeutic groups. In general the levels of DDD/tid increased as age increased, except for nucleosides and nucleotides where the situation was the opposite. The volumes of SSRIs as well as nucleosides and nucleotides were higher among females than males, whereas the volumes of ACE-inhibitors and statins were higher among males than among females.

In two groups for long-term use, ACE-inhibitors and statins, there were few differences in how the sales pattern developed for substitutable and non-substitutable drugs between different age and gender groups (data not shown). The sales pattern of substitutable and non-substitutable SSRIs varied between different age and gender groups (data not shown). For females aged 15–44, 45–64 and 65–74 years the volumes of non-substitutable SSRIs more than doubled from the start of the study period until the reform was implemented, and thereafter levelled out. A slight increase was seen for males of corresponding age but not as manifested as for females. Sales of substitutable increased somewhat in these age categories after the introduction of the reform.

For nucleosides and nucleotides the volumes of non-substitutable drugs were higher than the volumes of substitutable drugs in younger age groups (data not shown). However, at the end of the study period volumes of substitutable nucleosides and nucleotides were at the same level as non-substitutable nucleosides and nucleotides in the oldest age group. Volumes of both non-substitutable and substitutable nucleosides and nucleotides increased in the three youngest age groups. This was seen for both genders. For both males and females over 75 years of age, volumes of non-substitutable nucleosides and nucleotides remained unchanged, whereas volumes of substitutable nucleosides and nucleotides increased.

For PPIs the sales patterns of substitutable and non-substitutable drugs were similar in different age and gender groups (data not shown). However, when volumes of each substance were examined, the sales pattern for esomeprazole varied. Relatively speaking, esomeprazole was more frequent in younger people than in older age groups. There were no differences between the genders.

## Discussion

The present study showed a proportionally larger increase in the volumes of dispensed substitutable pharmaceuticals compared with non-substitutable pharmaceuticals, since the introduction of generic substitution. Thus sales of substitutable drugs have increased proportionally more than non-substitutable drugs, which indicates that lowered prices have contributed to an increase in the prescription of substitutable drugs. The changes were more marked in therapeutic groups where the patents expired during the implementation of the reform; however, this should be interpreted with caution, as this study used a descriptive approach. There were no differences between short- and long-term treatments where expiry of patents was close at hand. Differences in sales patterns were more often observed between different age groups than between the genders. Differences in sales patterns were not homogeneous across age and gender and between the selected therapeutic groups. There was a tendency that recently introduced non-substitutable pharmaceuticals were relatively more frequently sold in the youngest age groups.

The increased volumes of dispensed substitutable pharmaceuticals in the selected therapeutic groups indicated that prescribers facilitated the implementation of the reform, thus contributing to savings. This is in line with a previous study, reporting that substitution was seldom restricted by the prescriber [11]. The pronounced competition, resulting in reduced prices of many substitutable products, might have been an important contributor to the increased sales of these products. For example, in February 2006 the price of one of the most frequently sold packages of generic citalopram corresponded to 10–15% of the price of original brand citalopram before the reform. Reduced prices following the introduction of generic substitution have also been reported from other countries [5,6]. In Sweden, reduced prices, combined with the ongoing process of decentralisation of budget responsibility for reimbursed pharmaceuticals, might have provided incentives for the physicians to be more cost-conscious when prescribing. This is in line with studies of the British fundholding schemes that reported an increase in the prescription of generic products in fundholding practices, which contributed to lower costs per volume of prescribed pharmaceuticals [18,19]. Other factors that might have influenced sales patterns are recommendations from the county councils' Drug and Therapeutics Committees, DTC, discussions on safety issues of rather new pharmaceuticals, marketing as well as new studies on effects and safety of pharmaceuticals. Each county council has their own DTCs, and thus recommended drugs can vary within the country. In general DTCs revise their advice once a year. Many DTCs have changed their advice to recommend products that have recently become substitutable when the patent of a large product has expired.

In general the most dramatic changes are expected when patents expire, even when there is no substitution policy. However, the magnitude of these changes depends on a number of factors, such as policies that enable dispensing of generic alternatives, the prescribing habits of physicians, and incentives for patients to be cost-conscious. The marked effects seen for statins and PPIs were probably affected by the pronounced price competition for top-selling substances where the patent had expired during implementation of the reform. However, the design of this study does not allow for assessment of possible differences in comparison with a situation where there is no generic substitution. Price differences between originator and generic products were greater in the groups where the patent had expired recently. This seemed to be an important factor influencing sales patterns, as there were no differences between short- and long-term treatments with similar patent expiry times. There were also a larger number of generic alternatives in these groups than in groups where the patent had expired some years ago, which, together with DTC recommendations, might have contributed to the dramatic effects. For therapeutic groups where the patent of the substitutable substances had expired some years before the reform was introduced, ACE-inhibitors and nucleosides and nucleotides, changes in sales patterns related to the introduction of the reform were not as pronounced. However, on substance level there was a delayed increase in sales of non-substitutable substances in both groups. Altogether, these results are in accordance with a previous study which reported that the proportion of performed substitutions was highest in groups where the patent had expired close to the introduction of the reform and price differences were considerable [11]. The potential impact of DTC recommendations can be illustrated by the patent expiry of one of the nucleosides and nucleotides. The sales of substitutable nucleosides and nucleotides, i.e. aciclovir, started to increase in 2001, when the patent expired and cheaper products containing aciclovir became available. Several DTCs recommended the generic products which contributed to increased volumes of aciclovir.

It was somewhat surprising that the sales pattern of the two top-selling substitutable ACE-inhibitors (enalapril and ramipril) was similar to that seen for substitutable and non-substitutable drugs in other groups. Ramipril was similar to a non-substitutable substance since there were few substitutable products and little price competition until 2004, which was reflected by a higher price per dose compared with enalapril, where generic products were available when generic substitution was introduced. Another factor that might have affected sales patterns is the recommendations from the DTCs that varied between the three largest county councils, as well as over time. One recommended the use of ramipril, one recommended enalapril, whereas the third recommended both ramipril and enalapril.

### Strengths and limitations

All groups except nucleosides and nucleotides were among the five top-selling main drug groups during the study period regarding volume of DDD. Nucleosides and nucleotides were among the most widely sold anti-infective groups for short-term treatment fulfilling inclusion criteria. Three groups: PPIs, statins and SSRIs, were among the five groups that generated the highest expenditures between 2003 and 2005. Expenditure has decreased since the reform was introduced for several groups where the major products became substitutable, e.g. ACE-inhibitors and statins. Although the investigated groups are widely used, it should be noted that the results are not generalisable to all therapeutic groups comprising substitutable and non-substitutable pharmaceuticals. Sales patterns depend on the prevalence and nature of the disease in question, the characteristics of the drugs in the group, and also on when the patent expires and which products become substitutable.

A limitation of the study was that sales data comprising dispensed prescription medicines were used to reflect changes in prescribing patterns in this study. Not all prescribed medication is actually purchased by patients, and thus there is a gap between sales and prescribing data [20,21]. On the other hand, sales data provided full coverage of all dispensed prescription pharmaceuticals in Sweden over more than five years, with more than two years of baseline sales of the drugs before the introduction of the reform. We have no reason to believe that failure to purchase prescribed medication would differ with respect to substitutable and non-substitutable drugs, since both were equally covered by the PBS. Another limitation was that the data did not cover drugs provided to patients in hospitals, since these data are only registered in patient records. However, the selected pharmaceuticals are mainly used in outpatient care and thus data on dispensed prescription medicines provides a reliable picture. Comparisons of volumes across different age and gender groups were not adjusted for the epidemiology of the drug use, as the study used a descriptive approach. However, the level and distribution of volumes across age and gender were in accordance with previous studies [22-25]. No formal statistical analysis, such as time series analysis, was undertaken in this study. The main reasons for this were that the aim was to explore how sales patterns had developed rather than to analyse effects, and also that the data were not suitable for time series analysis. In order to reduce dilution, the sales of non-substitutable packages of substitutable substances were removed, although these constituted small volumes. Examples of non-substitutable packages include oral suspensions, ocular ointment and combination products. These products comprised rather small volumes of these substances, in general below 1%. An exception was fluvastatin, where a rather frequently prescribed strength of depot capsule was non-substitutable since no equivalent products were available.

## Conclusion

Since the introduction of generic substitution, there has been a proportionally larger increase in the volumes of dispensed substitutable drugs compared with the volumes of non-substitutable pharmaceuticals, indicating that the reform has contributed to an increase in sales of less expensive pharmaceuticals. This was especially pronounced for therapeutic groups where one or more major patents expired during the implementation of the reform. However, this study cannot assess whether this differed compared with patent expiry in a setting without generic substitution. This thus indicates that the reduction in prices might have affected the prescription of pharmaceuticals, contributing to increased sales of substitutable products, which in turn implies increased sales of less expensive equivalents.

## Competing interests

The author(s) declare that they have no competing interests.

## Authors' contributions

All authors participated in designing the study and discussed the findings. KA carried out data collection and data management and drafted the manuscript. MP participated in data management and revised the manuscript. PA revised the manuscript. AC took part in data collection and revised the manuscript. All authors read and approved the final manuscript.

## Pre-publication history

The pre-publication history for this paper can be accessed here:


